# Frequency-Modulated Wave Dielectrophoresis of Vesicles And Cells: Periodic U-Turns at the Crossover Frequency

**DOI:** 10.1186/s11671-018-2583-5

**Published:** 2018-06-07

**Authors:** Hiroshi Frusawa

**Affiliations:** School of Environmental Science & EngineeringKochi University of Technology, Tosa-Yamada, Kochi, 782-8502 Japan

**Keywords:** Dielectrophoresis, Frequency-modulated wave, The Clausius-Mossotti factor, Crossover frequency, Cell, Vesicle, Spectroscopy

## Abstract

We have formulated the dielectrophoretic force exerted on micro/nanoparticles upon the application of frequency-modulated (FM) electric fields. By adjusting the frequency range of an FM wave to cover the crossover frequency *f*_*X*_ in the real part of the Clausius-Mossotti factor, our theory predicts the reversal of the dielectrophoretic force each time the instantaneous frequency periodically traverses *f*_*X*_. In fact, we observed periodic U-turns of vesicles, leukemia cells, and red blood cells that undergo FM wave dielectrophoresis (FM-DEP). It is also suggested by our theory that the video tracking of the U-turns due to FM-DEP is available for the agile and accurate measurement of *f*_*X*_. The FM-DEP method requires a short duration, less than 30 s, while applying the FM wave to observe several U-turns, and the agility in measuring *f*_*X*_ is of much use for not only salty cell suspensions but also nanoparticles because the electric-field-induced solvent flow is suppressed as much as possible. The accuracy of *f*_*X*_ has been verified using two types of experiment. First, we measured the attractive force exerted on a single vesicle experiencing alternating-current dielectrophoresis (AC-DEP) at various frequencies of sinusoidal electric fields. The frequency dependence of the dielectrophoretic force yields *f*_*X*_ as a characteristic frequency at which the force vanishes. Comparing the AC-DEP result of *f*_*X*_ with that obtained from the FM-DEP method, both results of *f*_*X*_ were found to coincide with each other. Second, we investigated the conductivity dependencies of *f*_*X*_ for three kinds of cell by changing the surrounding electrolytes. From the experimental results, we evaluated simultaneously both of the cytoplasmic conductivities and the membrane capacitances using an elaborate theory on the single-shell model of biological cells. While the cytoplasmic conductivities, similar for these cells, were slightly lower than the range of previous reports, the membrane capacitances obtained were in good agreement with those previously reported in the literature.

**Electronic supplementary material:**

The online version of this article (10.1186/s11671-018-2583-5) contains supplementary material, which is available to authorized users.

## Background

The polarizability of an electrical phenotype is primarily due to the cell membrane and the cytoplasmic electrical properties that depend on the frequency of the applied electric field. Accordingly, individual cells can be identified by the differences in the dielectric spectra using noninvasive electrical techniques. The electrical techniques are currently competent for separating cells with useful phenotypes from unknown samples [1–15]. Compared with other separation methods, these offer the major advantage that cell modification by antibodies or adherence to foreign material is unnecessary, whereby the potential for cell damage or activation by these probes is avoided [1–16]. The characterization of the cellular dielectric properties has been performed mainly using either impedance spectroscopy [10, 12, 13] or alternating-current (AC) electrokinetics such as dielectrophoresis (DEP), traveling-wave DEP (twDEP), and electrorotation [1, 9, 15]. Among them, we focus on extending the AC-DEP method to develop a new method for dielectric characterization using the frequency-modulated (FM) waves instead of AC fields.

In general, the DEP occurs in an electric-field gradient that creates an electrokinetic force exerted on any polarizable object, charged or neutral, in the direction determined not only by the gradient vector, but also by the real part of the Clausius-Mossotti (CM) factor [1–15, 17–21]. For instance, we consider the DEP force induced by the AC electric field ***E***_AC_(***r***,*t*) whose space-time dependence is expressed as ***E***_AC_(***r***,*t*)=***A***(***r***) cos*θ*_AC_(***r***,*t*) using the amplitude vector *A*(***r***) and the phase *θ*_AC_(***r***,*t*). The AC-DEP force is generated by the spatial gradient of the amplitude (i.e., ∇***A***) multiplied by the real part of the CM factor, as mentioned above, whereas the spatial gradient of the phase (i.e., ∇*θ*_AC_) multiplied by the imaginary part of the CM factor creates the force of either twDEP or electrorotation, which therefore provides complementary information to the AC-DEP method in terms of the dielectric characterization [9, 15, 20, 21].

In this letter, we aim to formulate the DEP force induced by an FM field and compare the AC- and FM-DEP methods, so that neither the AC nor the FM field considers the spatial dependence of the phase; therefore, we will set *θ*_AC_(*t*)=2*πf*_AC_*t* in proportion to the applied frequency *f*_AC_. A significant feature of the AC-DEP is that the force direction as well as its strength depends on *f*_AC_. Most notably, the force direction is reversed at crossover frequency *f*_AC_=*f*_*X*_ due to the change in sign of the real part of the CM factor, which has been found available for the dielectric characterization using AC-DEP [1–15].

The frequency dependence of the AC-DEP force has also made the following manipulations possible [1–15, 22–31]: electrically controllable trapping, focusing, and translation of colloidal particles, as well as the fractionation and characterization of living and/or dead cells. Conventional systems for the dielectrophoretic assembly and/or manipulation of colloidal particles have often made use of microfabricated electrodes between which the AC electric field has been applied to colloidal suspensions, benefiting from recent rapid advances in the fabrication of integrated semiconductor devices [24–30]. This technology, offering noncontact manipulation, is currently being integrated with a variety of lab-on-a-chip systems that provide the advantage of accurate and repeatable handling. Nevertheless, the on-chip electrodes that create high-intensity spots in AC fields are incapable of changing their positions independently of the sample holder, in contrast to the laser focus that can be freely positioned in the optical manipulation. It follows from the limitation of on-chip systems that the previous DEP methods have presented some difficulty and complexity in performing the types of operations for which optical tweezers are suitable. A candidate method to overcome these difficulties is optical image-driven DEP [32].

**Fig. 1:**
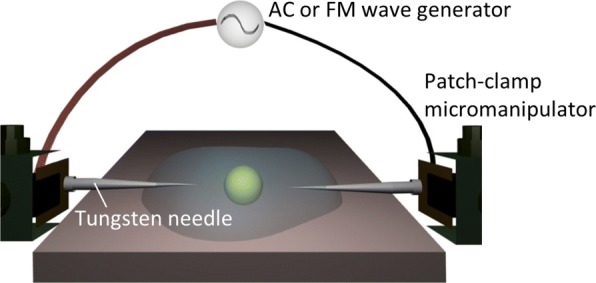
Experimental setup. A schematic of the dielectrophoretic manipulation system illustrating the AC or FM electric field applied to a target particle via a pair of electrode needles controlled by patch-clamp micromanipulators

Here, we adopted, as a simpler alternative, one of the electronic tweezers techniques [22, 23, 33–38] for on-demand dielectrophoretic assembly and/or manipulation without an optical apparatus (see Fig. 1). As seen from Fig. 1, our plug-in style system uses a pair of microelectrode needles that are controlled by micromanipulators for applying the external electric fields in a colloidal suspension. The electrode probes were not fixed but rather movable in colloidal suspensions owing to their plug-in style. There remains, however, a significant requirement for the practical use of dielectric characterization: the duration for which an electric field is applied to the cells surrounded by salty electrolytes should be minimized. For instance, the AC-DEP method involves the use of interdigitated comb-like electrodes that are embedded in a microfluidic system so that the AC fields of various frequencies can be simultaneously applied in a cell suspension [24–30]. While such refined on-chip systems have been found relevant for the dielectric characterization, the multielectrode-pair technique is inapplicable to the single-electrode-pair system which has been often used in the electronic tweezers techniques [22, 23, 33–38].

To accomplish simultaneous multifrequency measurements using the single-electrode-pair system (Fig. 1), the change in the applied electric field should be investigated. In this letter, we address the availability of time-varying DEP due to an FM wave (FM-DEP) of the following form:

1 $$ \boldsymbol{E}(\boldsymbol{r},t) =\boldsymbol{A}(\boldsymbol{r})\cos\theta(t),   $$

where the phase *θ*(*t*) of the FM wave is related to the instantaneous frequency *f*(*t*) as 2*πf*(*t*)=*dθ*(*t*)/*dt* and

2 $$ f(t)=f_{c}+\Delta f\cos\left(2\pi f_{m}t \right),   $$

with *f*_*m*_ denoting the modulation frequency. We use the wide band FM satisfying that *Δf*/*f*_*m*_≫1, so that the conditions of *f*_*m*_/*f*(*t*), *f*_*m*_/*f*_*c*_, *f*_*m*_/*Δf*≪1 will be referred to as the wide band limit (WBL) in the theoretical formulation given below.

In this letter, particular attention is paid to the relationship between the characteristic frequency of *f*_*X*_ and the trajectory of FM-DEP. In the next section, we describe both the materials used and details of the plug-in system for inducing FM-DEP. The third section provides the results and discussion that consists of four parts. First, we investigate the details of repeating U-turns of a single leukemia cell by quantifying the reciprocating trajectory, whose periodicity is explained by the modulation frequency *f*_*m*_, or the periodical oscillation of *f*(*t*) given by Eq. (2). Next, we explain the reciprocating trajectory theoretically by deriving the time-varying dielectrophoretic force that modulates according to the instantaneous frequency *f*(*t*) of the FM field satisfying the WBL condition. The obtained form of the dielectrophoretic force provides the equation that determines the *f*_*X*_ from the observed U-turns. Third, we measure the magnitude of the dielectrophoretic force on a multilamellar vesicle (MLV) that was attached to an electrode needle due to the attraction of the AC-DEP. The frequency dependence of force was fitted using the spectral equation that was determined from the real part of the CM factor, so that *f*_*X*_ was determined as the characteristic frequency at which the attractive force due to AC-DEP vanishes. Because the FM-DEP method also gives *f*_*X*_ by analyzing the reciprocating trajectory of an MLV, we assess the extent of coincidence between the crossover frequencies evaluated from AC- and FM-DEPs. Finally, both of the cytoplasmic conductivities and the membrane capacitances of three kinds of cell were evaluated from *f*_*X*_ as an increasing function of the solution conductivity, and the obtained values were compared with those reported in the literature.

## Methods

### Materials

For preparing multilamellar vesicles (MLVs), we used 1,2-dioleoyl-sn-glycero-3-phosphatidylcholine (DOPC) as lipids, purchased from Avanti Polar Lipids. The MLVs were obtained by the following procedure. The DOPC (1 mL, 20 mM) dissolved in chloroform/methanol (2:1 *v*/*v*) was dried with N_2_ gas, and the solvent was completely removed under a vacuum for more than 12 h. The thin film deposited on the glass vial due to the evaporation was rehydrated using deionized water and incubated at 25 °C for several hours.

Two cell lines used in the experiments were JKT-beta-del of human T cell leukemia (TL) line and CCRF-SB of human B cell leukemia (BL) line. Both kinds of TL and BL cells were used after 1 week incubation in a humidified incubator that contains 5 *%* CO_2_ at 37, so that we had the cell concentrations within the range of 0.5×10^6^ to 1×10^6^ cells/mL. The RPMI 1640 medium for the cell culture was supplemented with 10% fetal brovine serum and 100 mM sodium pyruvate. The cells were sedimented by centrifugation at 370*g* for 3 min twice so that the cells could be purely resuspended in 1 ml of the RPMI 1640 medium prior to the pipetting. The obtained cell suspensions were further diluted using the isotonic 200 mM sucrose solution in order to prepare for the solvent having a required conductivity.

We also used human red blood (RB) cells dispersed in the following suspensions. Freshly drawn whole blood samples were obtained from healthy volunteers in their early twenties. The cells, suspended in a mixture of the RPMI 1640 medium and hematocrit of 3.1%, were diluted using the isotonic 200 mM sucrose solution in order to prepare for the solvent having a required conductivity as well as the above leukemia cells. All of the dielectrophoretic experiments using the human RB cells were finished within 10 mins after drawing the whole blood samples.

### Experimental Setup

The conductivities of the cell suspensions were measured using a conductivity meter (SevenMulti, Mettler-Toledo, Columbus, OH, USA). A schematic of the plug-in system used is shown in Fig. 1. An external electric field with an AC or FM wave was applied via an arbitrary waveform generator (Agilent 33220A, Agilent Technologies, Santa Clara, CA, USA) with a current amplifier (F30PV, FLC Electronics, Partille, Sweden) to which plug-in-type microelectrodes were connected. The microelectrodes comprised tungsten needles with a tip diameter of 0.5 *μ*m that were independently controlled by two sets of patch-clamp micromanipulators (NMN-21, Narishige, Setagaya-ku, Tokyo, Japan). In all of the experiments that follow, we maintained the tip separation at 100 *μ*m when applying the external fields to the above suspensions, and the maximum magnitude was set to be 0.5 kV/cm. The needle pair was inserted into a sample drop mounted on the inverted optical microscope (TE2000-U, Nikon, Minato-ku, Tokyo, Japan), and the optical micrographs were obtained using a CCD camera (Retiga Exi, QImaging, Surrey, British Columbia, Canada) with a frame rate of 25 fps; incidentally, it was confirmed that the frequency resolution of FM waves due to the frame rate was always within the error bars for each data. A 50- *μ*l drop of suspension was mounted on the sample stage of the inverted optical microscope, whose temperature was maintained at 25 °C using a heat controller.

The plug-in technique allows the simple system to perform various noncontact manipulations of a single cell, such as pushing it into a narrow channel without any contact and orienting it toward the desired direction. Although it is often necessary to treat the cells in an isotonic solution with salt, it is easiest to implement the above DEP manipulations of cells surrounded by deionized water. In Additional file 1: Movies S1 to S3, the plug-in system induced the AC-DEP of diatom cells suspended in deionized water. We can see from Additional file 1: Movies S1 to S3 that an anisotropic diatom cell dispersed in salt-free water was manipulated like a post-it tag by a pair of microelectrodes between which the AC electric field (1 kV/cm) was applied. The noncontact operations consist of three steps: (i) a target cell was first rotated in parallel to a glass wall positively charged by the combination of dipole alignment at a frequency of 30 kHz and positional change of each microelectrode (Additional file 1: Movie S1), (ii) we subsequently changed the frequency to 100 kHz for pushing it toward the wall to fix the request cell with negative charges on the glass surface electrostatically (Additional file 1: Movie S2), and (iii) the AC frequency was adjusted to 20 MHz for inducing the AC-DEP in the opposite direction, so that the electrostatically attached cell could be pulled out (Additional file 1: Movie S3).

## Results and Discussion

### Experimental Observation of a Leukemia Cell Experiencing FM-DEP

Our plug-in microelectrodes (see Fig. 1) allow the electric field to be applied to the particles floating far above the sample substrate, which is of practical use for selecting appropriate cells. For instance, Additional file 1: Movie S4 shows that the microelectrode pair was controlled to approach a floating triangular diatom cell to which we applied the AC electric field with its frequency jumping between 100 and 500 kHz at intervals of 0.5 s. In Additional file 1: Movie S4, we see the triangular cell bouncing on a microelectrode due to the frequency jumping as a preliminary result in advance of the following manipulation using the FM-DEP.

**Fig. 2:**
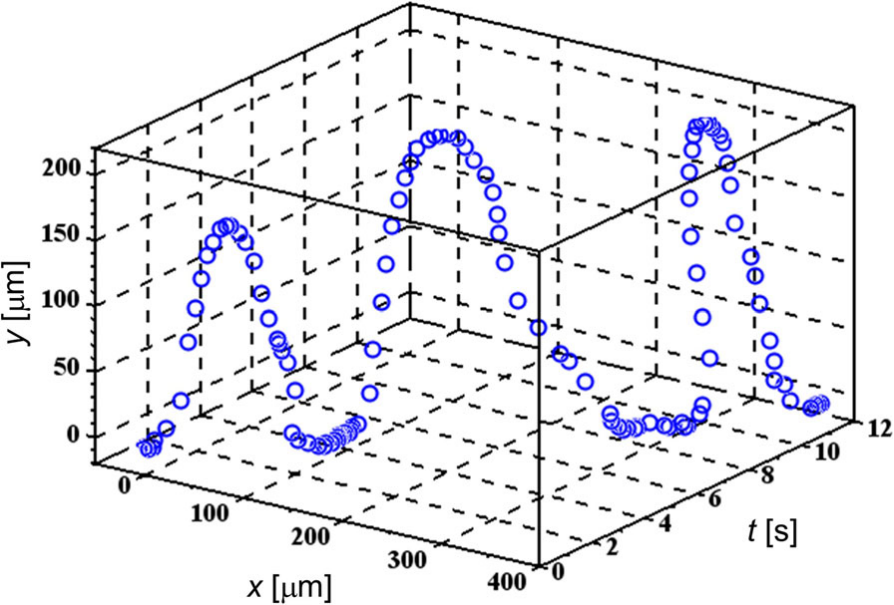
3D trajectory of a targeted TL cell. Periodic U-turns due to the frequency modulation are demonstrated for the TL cell undergoing the FM-DEP

Additional file 1: Movies S5 and S6 shows typical behaviors of several TL cells experiencing the FM-DEP, which are similar to those of mammalian cells manipulated by an electronic tweezers using a single electrode AC-DEP [36]. Figure 2 depicts one of the periodic trajectories using the 3-D plot of (*x*,*y*) along the *t* axis, where a relative coordinate of (*x*,*y*) is assigned to the temporary cell position with the origin of (0, 0) located at a specific point on a microelectrode needle for extracting the cell-electrode configuration. While the *x* axis represents the tangent to the electrode surface at (0, 0), the *y* axis, perpendicular to the tangent, mainly reflects the projection of the periodic U-turns explained below. In Fig. 2, we selected a floating TL cell to which we applied the FM electric field with its modulation frequency *f*_*m*_ set to be *f*_*m*_=0.25 Hz in the range of 200 kHz ≤*f*(*t*)≤ 3 MHz. Because we have that *Δf*/*f*_*m*_, *f*(*t*)/*f*_*m*_<10^−5^, the WBL condition actually holds, as mentioned after Eq. (2).

It is found from Additional file 1: Movies S5 and S6 as well as Fig. 2 that the periodic trajectory is constituted by three parts of leaving, approaching, and staying on the microelectrode: (i) the cell leaves the microelectrode, (ii) it approaches the microelectrode after doing a U-turn, and (iii) it stays on the microelectrode surface. The cell is often unable to return to the same position on the microelectrode surface because of the solvent flow, which is not only observed in Additional file 1: Movie S6, but is also represented by the U-turns with the cell migrating in the *x* direction in Fig. 2. Despite the interference with the solvent flow, it is possible to distinguish the moments when the cell starts to leave the microelectrode surface and does the U-turn in the periodic trajectory, respectively. Accordingly, we can see from Fig. 2 that these U-turns are repeated at intervals of 4 s in coincidence with the modulation frequency of 0.25 Hz, or the 4-s period of the instantaneous frequency *f*(*t*).

### Theoretical Study on the FM-DEP

**Fig. 3:**
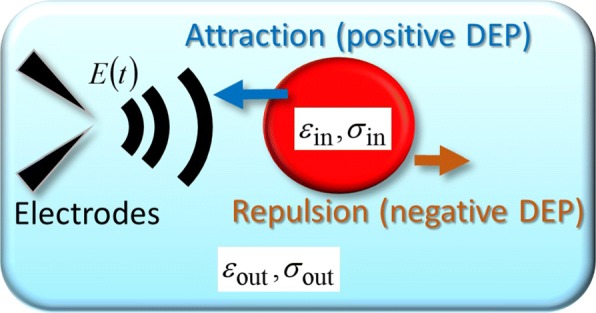
Theoretical model. Schematic representation of the FM-DEP force exerted on a cell that is modelled as a homogeneous sphere model having the permittivity and conductivity of *ε*_in_ and *σ*_in_, respectively. The sphere is surrounded by electrolyte medium with its permittivity and conductivity of *ε*_out_ and *σ*_out_, respectively. The homogeneous sphere model is the simplification of the spherical single shell model that regards the cell as a smeared-out cytoplasm surrounded by a membrane [9]

To explain the experimental trajectories, including the periodic U-turns, we consider a spherical object as a simplified model of a single cell, to which an arbitrary time-varying electric field ***E***(***r***,*t*) is applied. Figure 3 shows a schematic of the time-dependent DEP force acting on a spherical object [9]. As shown in Fig. 3, the permittivity and conductivity inside a spherical object are represented by *ε*_in_ and *σ*_in_, respectively, and the subscript “out,” such as *ε*_out_ and *σ*_out_, denotes the outside. In general, ***F***_DEP_(***r***,*t*) is related to the induced dipole moment ***p***(***r***,*t*) as [17–19]

3 $$\begin{array}{@{}rcl@{}} \boldsymbol{F}_{\text{DEP}}(\boldsymbol{r},t)&=&\left\{\boldsymbol{p}(\boldsymbol{r},t)\cdot \nabla\right\}\boldsymbol{E}(\boldsymbol{r},t), \end{array} $$

4 $$\begin{array}{@{}rcl@{}} \boldsymbol{p}(\boldsymbol{r},t)&=&4\pi R^{3}\epsilon_{\text{out}}K_{H}\left\{\boldsymbol{E}(\boldsymbol{r},t)+\frac{\tau}{\Delta\tau} \widetilde{\boldsymbol{E}}(\boldsymbol{r},t)\right\},  \end{array} $$

5 $$\begin{array}{@{}rcl@{}} \widetilde{\boldsymbol{E}}(\boldsymbol{r},t)&=&\frac{1}{\tau}\int_{0}^{tds}\,\boldsymbol{E}(\boldsymbol{r},t-s)e^{-s/\tau}, \end{array} $$

where *K*_*H*_ and *Δτ* are defined as follows: *K*_*H*_=(*ε*_in_−*ε*_out_)/(*ε*_in_+2*ε*_out_), and $\Delta \tau ^{-1}=\tau _{0}^{-1}-\tau ^{-1}$ using the radius *R* of the spherical object and two characteristic times of *τ*_0_=(*ε*_in_−*ε*_out_)/(*σ*_in_−*σ*_out_) and *τ*=(*ε*_in_+2*ε*_out_)/(*σ*_in_+2*σ*_out_).

Substituting the AC electric field ***E***_AC_(***r***,*t*)=***A***(***r***) cos(2*πf*_AC_*t*) into Eqs. (3) to (5), we obtain the mean DEP force <***F***_DEP_(***r***,*t*)> that has been averaged over cycles of the AC field [9, 15, 20]:

6 $$\begin{array}{@{}rcl@{}} \left<\boldsymbol{F}_{\text{DEP}}\right>
&=&4\pi R^{3}\epsilon_{\text{out}}K_{H}\left[ \left<\boldsymbol{E}\cdot \nabla\boldsymbol{E}\right>
+\frac{\tau}{\Delta\tau}\left<\widetilde{\boldsymbol{E}}\cdot \nabla\boldsymbol{E}\right>
\right]\\ &=&\chi(f_{\text{AC}})\nabla\boldsymbol{A}^{2}_{\text{RMS}},  \end{array} $$

where ***A***_RMS_ denotes the root mean squared (RMS) vector satisfying that $\boldsymbol {A}_{\text {RMS}}^{2}=\boldsymbol {A}^{2}/2$, and *χ*(*f*_AC_)≡2*πR*^3^*ε*_out_Re[*K*(*f*_AC_)] depends on the applied frequency *f*_AC_ due to Re[*K*(*f*_AC_)], the real part of the CM factor [9, 15, 20]:

7 $$\begin{array}{@{}rcl@{}} \chi(f_{\text{AC}})=\frac{2\pi R^{3}\epsilon_{\text{out}}}{1+(2\pi f_{\text{AC}}\tau)^{2}} \left\{ K_{L}+(2\pi f_{\text{AC}}\tau)^{2}K_{H} \right\},  \end{array} $$

where *K*_*L*_=(*σ*_in_−*σ*_out_)/(*σ*_in_+2*σ*_out_) and *K*_*H*_, defined above, correspond to the real CM values in the low- and high-frequency limits, respectively, and these limiting values, *K*_*L*_ and *K*_*H*_, need to have opposite signs so that *f*_*X*_ defined by *χ*(*f*_*X*_)=0 may exist [9, 15, 20].

Equations (6) and (7) indicate that the AC electric field creates the DEP force whose direction depends on the applied frequency *f*_AC_ through *χ*(*f*_AC_) given by Eq. (7), which explains the bouncing diatom cell in Additional file 1: Movie S4 as follows (see also Fig. 3). When the applied frequency provides the plus sign of the real part of the CM factor (i.e., *χ*(*f*_AC_)>0), we can observe cells attracted toward the electrode needle tips (the positive DEP) on which the strength of the AC field, applied via a pair of electrode needles, is the largest. The sign of the real CM factor can be reversed to the negative at *f*_*X*_, the vanishing frequency of the real CM factor (i.e., *χ*(*f*_*X*_)=0), where we have the zero dielectrophoretic force as found from Eq. (6). In the negative sign of the CM factor (i.e., *χ*(*f*_AC_)<0), individual colloids are repelled from the electrode needle pair (the negative DEP). The triangular diatom cell in Additional file 1: Movie S2 bounced because of the opposite direction of the AC-DEPs induced by the AC fields with their frequencies of 100 and 500 kHz; combining Eq. (6) and the dielectrophoretic directions observed, we find that *χ*(100 kHz)>0 and *χ*(500 kHz)<0.

Next, we consider the FM-DEP by plugging the phase given by Eqs. (1) and (2) into Eqs. (3) to (5). As proved in Additional file 2, the WBL condition of the FM wave validates the approximate form of the integration in Eq. (5), thereby providing

8 $$ \left<\widetilde{\boldsymbol{E}}\cdot \nabla\boldsymbol{E}\right>
=\frac{1}{1+\{2\pi f(t)\tau\}^{2}}\left(\frac{\nabla\boldsymbol{A}^{2}_{\text{RMS}}}{2} \right),  $$

which becomes the same form as that of the AC-DEP when the time-dependent frequency *f*(*t*) is replaced by a constant frequency of *f*_AC_. We thus obtain the limiting form of the mean DEP force <***F***_DEP_(***r***,*t*)> that has been averaged over cycles of *θ*(*t*) in the FM field (see Eqs. (A1), (A13) and (A14) in Additional file 2):

9 $$\begin{array}{@{}rcl@{}} \left<\boldsymbol{F}_{\text{DEP}}(\boldsymbol{r},t)\right>
=\chi\{f(t)\}\nabla\boldsymbol{A}^{2}_{\text{RMS}},  \end{array} $$

being of a similar form to Eq. (6) for the AC-DEP. The difference is whether or not the coefficient of *χ*{*f*(*t*)} depends on *t* through *f*(*t*), which changes cyclically according to the frequency modulation with the period of *T*_*m*_=1/*f*_*m*_.

**Fig. 4:**
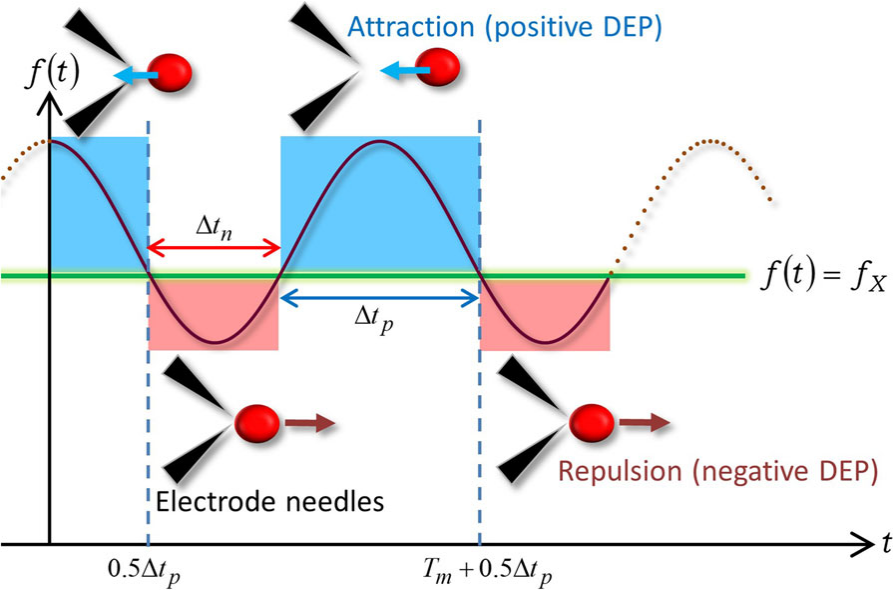
Force direction related to the frequency modulation. An illustration of the periodic U-turns due to the FM wave with its time-dependent frequency of *f*(*t*) covering a crossover frequency *f*_*X*_

Based on the simple expression (9) of the FM-DEP, we illustrate with Fig. 4 the mechanism of the above U-turns due to the FM wave. Figure 4 shows a schematic of the DEP induced by the FM wave in the WBL when the range of *f*(*t*) covers the crossover frequency *f*_*X*_ such that *f*_*c*_−*Δf*≤*f*_*X*_≤*f*_*c*_+*Δf*. It is supposed in Fig. 4 that the frequency dependence of the real part of the CM factor, or *χ*{*f*(*t*)}, provides the alternate sign changes as follows: minus sign (*χ*{*f*(*t*)}<0) for *f*(*t*)<*f*_*X*_ and plus sign (*χ*{*f*(*t*)}>0) for *f*(*t*)>*f*_*X*_, which is the case with our experiments. The former period satisfying *f*(*t*)<*f*_*X*_ has a duration time, whereas the latter *f*(*t*)>*f*_*X*_ has been retained during the rest the period: one cycle is classified into two periods that are marked red and blue, respectively, in Fig. 4.

Similarly to the AC-DEP, Eq. (9) implies that the minus sign (*χ*{*f*(*t*)}<0) creates a repulsive DEP force between the cell and the microelectrodes while satisfying that *f*(*t*)<*f*_*X*_. As a result, the cell leaves the area around the micfroelectrode needle tips between which the magnitude of the electric field is the largest: the cell experiences the negative DEP during the red period of *Δt*_*n*_ in Fig. 4. At the instant *t*_*X*_ as a solution of *f*(*t*_*X*_)=*f*_*X*_, *χ*(*f*) vanishes, followed by the sign change into *χ*(*f*)>0 while *f*(*t*)>*f*_*X*_, and correspondingly the DEP force is switched to the attractive force at *t*_*X*_. After doing a U-turn at *t*_*X*_ due to the reversal in direction of the DEP force, the targeted cell starts to approach the microelectrode migrating in the opposite direction and is eventually trapped between the tips of the electrode needles or attached on one of the electrodes: the cell experiences the positive DEP during the blue period of *Δt*_*p*_ in Fig. 4. Figure 4 indicates that the cycle of leaving, approaching and staying on the microelectrode should be repeated with the modulation period of *T*_*m*_, in agreement with Fig. 2: *Δt*_*n*_+*Δt*_*p*_=*T*_*m*_. The dielectrophoretic mechanism depicted in Fig. 4 can thus explain the periodic U-turns observed in Additional file 1: Movies S5 and S6 as well as Fig. 2.

Let us consider the periodic solution of the equation, *f*(*t*_*X*_)=*f*_*X*_. As seen from Fig. 4, *t*_*X*_ is expressed as *t*_*X*_=*nT*_*m*_+0.5*Δt*_*p*_=*nT*_*m*_+0.5(*T*_*m*_−*Δt*_*n*_) using an integer of *n*=0, ±1, ±2,⋯, which further reads

10 $$ 2\pi f_{m} t_{X}=(2n+1)\pi-\pi f_{m}\Delta t_{n}.   $$

Substituting Eq. (10) into Eq. (2), we have for *n*=0 that

11 $$ f_{X}=f_{c}-\Delta f\cos\left(\pi f_{m}\Delta t_{n}\right),   $$

clarifying that the FM-DEP method determines the crossover frequency if the duration time *Δt*_*n*_ from leaving the microelectrode to doing the U-turn can be measured precisely.

### Comparing Crossover Frequencies of a Single MLV Determined from the FM- and AC-DEPs

We investigated the experimental accuracy of Eq. (11). Experimentally, it is often necessary for the biological cells to be dispersed in an electrolyte. For MLVs, however, the use of deionized water is allowed during the preparation process of rehydration and dilution. We thus used the salt-free MLV suspension for comparing the crossover frequencies determined from both AC- and FM-DEPs.

The dielectrophoretic U-turns of a targeted MLV were induced by the FM wave in the range of 10 kHz ≤*f*(*t*)≤ 50 kHz (i.e., *f*_*c*_=30 kHz and *Δf*=20 kHz) with a setting that *f*_*m*_=0.1 Hz, and correspondingly the FM-DEP has a 10-s period. In the experiments, it takes less than 30 s to observe a few U-turns of the targeted MLV from leaving to approaching microelectrodes. From the trajectory, we obtained the mean leaving time that $\overline {\Delta t_{n}}=5.8\pm 0.2$ s. Because the WBL condition applies to the present experiment satisfying that *f*_*m*_/*Δf*/*f*_*m*_, *f*_,_*m*/*f*(*t*)<10^−5^, the crossover frequency was evaluated to be *f*_*X*_=35±1 kHz from substituting $\overline {\Delta t_{n}}=5.8\pm 0.2$ s into Eq. (11).

For comparison, we made use of the programmable manipulator in the AC-DEP method that tries to evaluate the crossover frequency of the same targeted MLV to which the sinusoidal electric field with a frequency in the range of 30 to 100 kHz was applied via the electrode needle pair for inducing the AC-DEP. Because the programmable manipulator carries the electrode needle pair at a constant speed in one direction, we can measure the dielectrophoretic force similarly to the laser-trapping experiments [39]. Attaching the MLV on an electrode tip that undergoes uniform linear motion, not only the AC-DEP force but also the hydrodynamic force caused by the one-dimensional motion are exerted on the MLV. With the gradual increase of electrode velocity, *F*_DEP_ eventually becomes smaller than the hydrodynamic force. As a result, the MLV initially attached to the moving electrode, owing to the DEP attraction, is desorbed by the hydrodynamic force. Defining the critical value, *v*_*c*_, by the maximum velocity value of the microelectrode pair prior to the desorption, the force balance equation between the DEP and hydrodynamic forces reads [39]

12 $$ F_{\text{DEP}}(f_{\text{AC}})=6\pi\eta R v_{c},   $$

where *F*_DEP_(*f*_AC_)***e***≡<***F***_DEP_> with the unit vector ***e*** defined by $\boldsymbol {e}=\nabla {\boldsymbol {A}}^{2}_{\text {RMS}}/|\nabla {\boldsymbol {A}}^{2}_{\text {RMS}}|$, *η* the water viscosity at 25 °C and 2*R* the diameter of the MLV.

Additional file 1: Movies S7 and S8 demonstrates the force measurement using the above AC-DEP method at the applied frequency of *f*_AC_= 60 kHz. In Additional file 1: Movie S7, the velocity of the electrode pair controlled by the programmed manipulator is 110 *μ*m/s, which is lower than *v*_*c*_; therefore, the MLV remains attached to one part of the electrode pair owing to the dielectrophoretic attraction. Additional file 1: Movie S8, on the other hand, shows the higher electrode speed of 120 *μ*m/s, under which the dielectrophoretic force becomes smaller than the hydrodynamic force that is exerted on the MLV, thereby desorbing the MLV from the electrode. Accordingly, *v*_*c*_ is evaluated to be 110 *μ*m/s ≤*v*_*c*_≤ 120 *μ*m/s, and we can calculate *F*_DEP_(60 kHz) using Eq. (12).

**Fig. 5:**
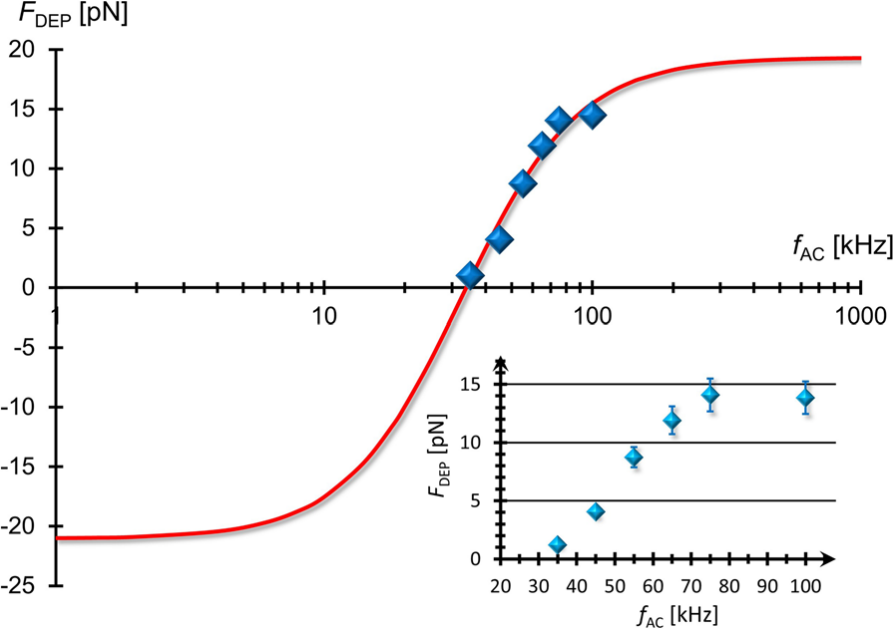
Frequency dependence of *F*_DEP_. The FM-DEP force (*F*_DEP_) as a function of external frequency (*f*_AC_) of applied AC field where *F*_DEP_ has been evaluated from Eq. (12), the balance equation between the FM-DEP and hydrodynamic forces exerted on a single MLV. It can be seen that *F*_DEP_ is increased and saturated as *f*_AC_ is higher, reflecting a typical behavior of the relaxation spectrum of the real CM factor. The solid line represents the best-fit result of Eq. (13)

We can determine *f*_*X*_ from the experimental results of *F*_DEP_ at various external frequencies. Figure 5 shows the frequency dependence of *F*_DEP_, indicating that the DEP force experienced by the MLVs was reduced by lowering the applied frequency. It is found from Eqs. (6) and (7) that the fitting function of *F*_DEP_(*f*_AC_) can be expressed as

13 $$ F_{\text{DEP}}(f_{\text{AC}})=\frac{L+(2\pi f_{\text{AC}}\tau)^{2}H}{1+(2\pi f_{\text{AC}}\tau)^{2}},   $$

implying that

14 $$ f_{X}=\frac{1}{2\pi\tau}\sqrt{-\frac{L}{H}}.   $$

Equation (13) is depicted by the solid line in Fig. 5 that has been fitted to the experimental data using the best-fit results of three parameters: *L*=−21.02 pN, *H*=19.03 pN, and *τ*=4.9 *μ*s. Substituting these results into Eq. (14), we evaluate that *f*_*X*_= 34.15 kHz, which coincides with the result of *f*_*X*_=35±1 kHz evaluated from the FM-DEP method. The FM-DEP method is thus validated in terms of the consistency with the direct force measurement using the AC-DEP method.

### Conductivity Dependencies of the Crossover Frequencies for Biological Cells

Let us return to the dielectrophoretic U-turns of biological cells mentioned in Fig. 2 to assess the practical reliability of the crossover frequencies when the FM-DEP method is applied to cell suspensions. Recently, an elaborate theory [40] has investigated, in more detail than before, the relationship between the homogeneous sphere model (see Fig. 3) and the single-shell model where the inner structure of cell is represented by a smeared-out cytoplasm surrounded by a membrane. As a result, the relation between *f*_*X*_ and the suspension conductivity *σ*_out_ has been formulated using radius *R* of a cell, membrane capacitance *C*_*m*_, and cytoplasmic conductivity *σ*_cyt_ [40]:

15 $$ f_{X}=\frac{1}{\sqrt{2}\pi {RC}_{m}}\left(\sigma_{\text{out}}-\frac{1}{2\sigma_{\text{cyt}}} \sigma_{\text{out}}^{2} \right)+f_{X0},   $$

where *f*_*X*0_ is the extrapolated value to the crossover frequency at *σ*=0 mS/m and will be treated as a fitting parameter herein. The elaborate treatment adds the squared term, the second term on the right hand side of Eq. (15), to the conventional linear relation which has mainly been used for evaluating *C*_*m*_ from *f*_*X*_ [40–45]. Theoretically, it has still been claimed [40] that Eq. (15) is valid within a lower range of *σ*_out_ such that *σ*_out_<10 mS/m; however, it should be better to include the squared term in the evaluation of *C*_*m*_, considering that our range of *σ*_out_ is relatively high compared with previous results in the range of 10 mS/m ≤*σ*_out_≤ 100 mS/m [40–45]. Hence, we determined *σ*_cyt_ as well as *C*_*m*_ from fitting Eq. (15) to the experimental results of *f*_*X*_ as an increasing function of *σ*_out_.

There are three kinds of biological cell used: TL and BL cells of human leukemia and RB cells of three human volunteers. In all the experiments using any species of cell, the conductivities were within the range of 60 to 160 mS/m, and the modulation frequency was set to be 0.25 Hz. Regarding the instantaneous frequency, most of the experiments adopted the range from 100 to 1.5 MHz (i.e., *f*_*c*_= 800 kHz and *Δf*=700 kHz); exceptionally for leukemia cells, the frequency range was extended to 50 kHz ≤*f*(*t*)≤1550 kHz (i.e., *f*_*c*_=800 kHz and *ΔfX*=750 kHz) in the conductivity range of 60 mS/m≤*σ*≤80 mS/cm because *f*_*X*_ in this *σ*-range has been found to be lower than 100 kHz, and we were unable to observe the DEP U-turns in the range of 100 kHz ≤*f*(*t*)≤1500 kHz. Both of these frequency sets satisfy the WBL condition of *Δf*/*f*_*m*_, *f*(*t*)/*f*_*m*_<10^−5^ as before.

Each time we measured the leaving times of cells dispersed in a suspension, we looked for an appropriate spot at which a few cells having a similar size could simultaneously experience the FM-DEP above the substrate, and the microelectrode tips were placed at the measurable position using the micromanipulator. We continued such scanning inside the cell suspensions until the FM-DEP trajectories of 10 cells were collected in total at a couple of appropriate positions. For each kind of cell, the measurement of 10 cells was repeated twice using different drops of the same cell suspension. As mentioned, it is indispensable for the implementation of the FM-DEP measurement at each spot to suppress the electrically induced solvent flows as much as possible. Hence, we traced only two cycles of the U-turn path so that the duration time of applying the electric field could be adjusted to be less than 10 s, and, correspondingly, the leaving time of each cell is given as the average of each trajectory, including the two U-turns. The mean leaving time $\overline {\Delta t_{n}}$ of each cell suspension is thus obtained from averaging the leaving times of 20 cells. Particularly for human RB cells, we further averaged three sets of the mean crossover frequencies obtained for three RB cell suspensions of three human beings, supposing that cells of the same species are similar in *C*_*m*_ and *σ*_cyt_ as well as in *R*. The two-step averaging of *Δt*_*n*_ will be denoted by $\left <\overline {\Delta t_{n}}\right >$. Substituting into Eq. (10) the experimental data of either $\overline {\Delta t_{n}}$ or $\left <\overline {\Delta t_{n}}\right >$, the mean crossover frequency <*f*_*X*_> was obtained.

**Fig. 6:**
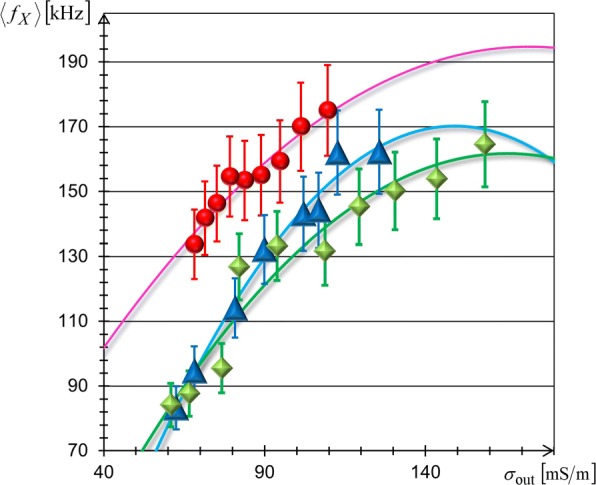
Conductivity dependences of crossover frequencies. Mean crossover frequency, <*f*_*X*_>, of TL cells (blue triangles), BL cells (green diamonds), and RB cells (red circles) varying with increase of solution conductivity *σ*_out_. The best-fit results of Eq. (15) are delineated by the solid lines

Figure 6 shows the *σ*_out_-dependencies of <*f*_*X*_> measured for the above three kinds of biological cells using the FM-DEP method. The solid lines in Fig. 6 depict the best-fit results of Eq. (15). We evaluated *C*_*m*_ and *σ*_cyt_ from the best fitting of Eq. (15) into which the observed radii (*R*_obs_) were inserted. Table 1 lists the fitting results of *C*_*m*_ and *σ*_cyt_, where we used the observed radii of 10 *μm*≤ 2*R*_obs_≤ 15 *μ*m for TL and BL cells, and 7.5 *μm*≤ 2*R*_obs_≤ 10 *μ*m for RB cells in evaluating *C*_*m*_. It is to be noted from Table 1 that different species have different membrane capacitances, which are in good agreement with those reported in the literature [40–47]; the *C*_*m*_ values of RB cells with stationary whole blood samples from normal (healthy) donors are in excellent agreement with our value [46, 47], but are substantially higher than those of washed RB cells in isotonic buffered saline as noted in [47]. The best-fit results simultaneously provided cytoplasmic conductivities, which were consistently similar as seen from Table 1, but were slightly lower than the range of previous reports that 0.2 S/m ≤*σ*_cyt_≤1 S/m [40, 45, 48–51]. These results support that the FM-DEP method retains the practical reliability needed for the treatment of living cells.

**Table 1:** Comparison of membrane capacitances *C*_*m*_ and cytoplasmic conductivities *σ*_cyt_

Cell species	*C*_*m*_[mF/m^2^]	*σ*_*cyt*_[mS/m]
TL cells (JKT-beta-del)	10.8 ± 2.2	149
BL cells(CCRT-SB)	16.0 ± 3.2	166
RB cells (human whole blood)	31.3 ± 6.7	172

JKT-beta-del of human T cell leukemia (TL) line, CCRF-SB of human B cell leukemia (BL) line, and human red blood (RB) cells in freshly drawn whole blood have different membrane capacitances and similar cytoplasmic conductivities, according to the evaluations in Fig. 6

## Conclusions

Our theoretical treatment of the FM-DEP has mainly focused on the WBL condition. In this limit, we have proved theoretically that the direction of the FM-DEP force switches each time when the instantaneous frequency of the FM wave traverses the crossover frequency, thereby implying the periodic U-turns of micro/nanoparticles that undergo the FM-DEP. Two kinds of experiment have demonstrated the accuracy and reliability of *f*_*X*_ obtained from the observed trajectories of MLVs and cells using our formulation of the FM-DEP (Eqs. (9) and (11)): While the *f*_*X*_ evaluated from the FM-DEP of a single MLV coincides with that obtained from the force measurement of the same MLV experiencing AC-DEP, the conductivity dependencies of *f*_*X*_ provide the membrane capacitances of various cells that are in close agreement with the literature values. In other words, it has been validated theoretically and experimentally that the FM-DEP in the WBL limit can be mimicked by the time-varying AC-DEP induced by the AC wave with its frequency changing continuously according to the periodic function of *f*(*t*). The simple view applies to other electrokinetics, including the twDEP and the electrorotation by applying the FM wave that has the spatial dependence of the phase as well as the magnitude. The AC- and FM-DEPs are associated with the real part of the dielectric spectra (or the CM factor), whereas the electrokinetics due to the spatial gradient of the phase reflect the imaginary part of the CM factor as mentioned before. Therefore, the application of the FM wave to either twDEP or electrorotation will be required for completing the dielectric characterization (the dielectric spectroscopy, in general) using the electrokinetics.

We have treated microparticles such as MLVs and cells for the precise tracking of particle trajectories. In these experiments, sedimented particles as well as floating ones have been observed; we need to increase the magnitude of electric field for inducing the DEP of the sedimented particles which are likely to be aggregated. Accordingly, we have used the plug-in system for applying the FM wave to a targeted particle floating above the substrate.

It is promising to further develop the FM-DEP method for smaller particles with their sizes of submicron to nanoscale, such as dispersed carbon nanotubes, thereby opening up the possibility of real-time spectroscopy using the FM-DEP as described below. When we apply the FM wave to the smaller colloids using the on-chip systems whose electrode configuration is designed to create a constant gradient of the applied electric field, the time-varying velocity vector ***v***(*t*) of the FM-DEP caused by the time dependence of the FM-DEP force is ascribed to the variation in *χ*(*f*) (or the real part of the CM factor): it is found from Eqs. (9) and (12) that

16 $$ \boldsymbol{v}(t)=\frac{\nabla\boldsymbol{A}^{2}_{\text{RMS}}}{6\pi\eta R}\chi\{f(t)\}.  $$

Hence, measuring the velocity vector ***v***(*t*) of a submicron to nanoparticle could provide the frequency dependence of the real part of the CM factor directly, which would be nothing but the electrokinetic FM spectroscopy.

## Additional files

Additional file 1 Eight movie clips of an elongated diatom cell manipulated like a post-it tag without contact to a microelectrode pair (Movies S1–3), a triangular diatom cell reciprocated on a microelectrode due to AC-DEP (Movie S4), human T cell leukemia cells reciprocated on a microelectrode due to FM-DEP (Movies S5 and S6), and a multilamellar vesicle (MLV) attracted to a moving microelectrode (Movies S7 and 8). **Movie S1**: Rotating a diatom cell by non-contact manipulation using the combination of dipole alignment and positional change of each microelectrode (*alignment operation*). **Movie S2**: Pushing a diatom cell toward a glass wall by non-contact manipulation to fix the request cell with negative charges on the glass surface electrostatically (*paste operation*). **Movie S3**: Pulling off a diatom cell from the glass wall by non-contact manipulation (*peel-off operation*). **Movie S4**: Periodic U-turns of a diatom cell due to AC-DEPs induced by frequency jumps of applied sinusoidal electric fields. **Movie S5**: Periodic U-turns of leukemia cells due to FM-DEP. **Movie S6**: Periodic U-turns of another leukemia cell due to FM-DEP under solvent flow. **Movie S7**: Carrying an MLV attached to one part of the microelectrode pair moving with lower speed. **Movie S8**: Desorption of an MLV attached to one part of the microelectrode pair moving with higher speed. (ZIP 27000 kb)

Additional file 2 Derivation of Eqs. (8) and (9) in the wide band limit (WBL). We provide the details in obtaining the approximate expression of Eqs. (8) and (9). Our focus is on clarifying how the integral in Eq. (5) is reduced to a simple form in the WBL, as seen from Eqs. (A2), (A3) and (A10). (PDF 33 kb)

## Abbreviations

AC: Alternating current
BL: B cell leukemia
CM: Clausius-Mossotti
DEP: Dielectrophoresis
DOPC: 1,2-Dioleoyl-sn-glycero-3-phosphatidylcholine
FM: Frequency modulated
MLV: Multilamellar vesicle
RB: Red blood
RMS: Root mean squared
TL: T cell leukemia
twDEP: Traveling wave dielectrophoresis
WBL: Wide band limit

## References

[1] Pethig R (2017). Dielectrophoresis: Theory, methodology and biological applications.

[2] Pethig R (2017). Where is dielectrophoresis (DEP) going?. J Electrochem Soc.

[3] Hughes MP (2016). Fifty years of dielectrophoretic cell separation technology. Biomicrofluidics.

[4] Dash S, Mohanty S (2014). Dielectrophoretic separation of micron and submicron particles: a review. Electrophoresis.

[5] Jubery TZ, Srivastava SK, Dutta P (2014). Dielectrophoretic separation of bioparticles in microdevices: a review. Electrophoresis.

[6] Gascoyne PR, Shim S, Noshari J, Becker FF, StemkeHale K (2013). Correlations between the dielectric properties and exterior morphology of cells revealed by dielectrophoretic fieldflow fractionation. Electrophoresis.

[7] Gagnon ZR (2011). Cellular dielectrophoresis: applications to the characterization, manipulation, separation and patterning of cells. Electrophoresis.

[8] Valero A, Braschler T, Renaud P (2010). A unified approach to dielectric single cell analysis: Impedance and dielectrophoretic force spectroscopy. Lab Chip.

[9] Pethig R (2010). Dielectrophoresis: status of the theory, technology, and applications. Biomicrofluidics.

[10] Sun T, Morgan H (2010). Single-cell microfluidic impedance cytometry: a review. Microfluid Nanofluid.

[11] Gascoyne PR, Noshari J (2009). Anderson TJ, Becker FF. Isolation of rare cells from cell mixtures by dielectrophoresis. Electrophoresis.

[12] Morgan H, Sun T, Holmes D, Gawad S, Green NG (2006). Single cell dielectric spectroscopy. J Phys D Appl Phys.

[13] Suehiro J, Hamada R, Noutomi D, Shutou M, Hara M (2003). Selective detection of viable bacteria using dielectrophoretic impedance measurement method. J Electrost.

[14] Gascoyne PR, Vykoukal J (2002). Particle separation by dielectrophoresis. Electrophoresis.

[15] Hughes MP (2002). Nanoelectromechanics in engineering and biology.

[16] Weiz SM, MedinaSnchez M, Schmidt OG (2017). Microsystems for SingleCell analysis. Adv Biosys.

[17] Lo YJ, Lin YY, Lei U, Wu MS, Yang PC (2014). Measurement of the Clausius-Mossotti factor of generalized dielectrophoresis. Appl Phys Lett.

[18] Lo YJ, Lei U, Chen KY, Lin YY, Huang CC, Wu MS, Yang PC (2014). Derivation of the cell dielectric properties based on Clausius-Mossotti factor. Appl Phys Lett.

[19] Lei U, Lo YJ (2011). Review of the theory of generalised dielectrophoresis. IET Nanobiotechnol.

[20] Voldman J (2006). Electrical forces for microscale cell manipulation. Annu Rev Biomed Eng.

[21] Hughes MP (2000). AC electrokinetics: applications for nanotechnology. Nanotechnology.

[22] Frusawa H, Yoshii G (2015). Anisotropic micro-cloths fabricated from DNA-stabilized carbon nanotubes: one-stop manufacturing with electrode needles. Nanoscale Res Lett.

[23] Frusawa H, Manabe T, Kagiyama E, Hirano K, Kameta N, Masuda M, Shimizu T (2013). Electric moulding of dispersed lipid nanotubes into a nanofluidic device. Sci Rep.

[24] Li M, Li WH, Zhang J, Alici G, Wen W (2014). A review of microfabrication techniques and dielectrophoretic microdevices for particle manipulation and separation. J Phys D Appl Phys.

[25] Nakano A, Ros A (2013). Protein dielectrophoresis: advances, challenges, and applications. Electrophoresis.

[26] MartinezDuarte R (2012). Microfabrication technologies in dielectrophoresis applications: a review. Electrophoresis.

[27] Cetin B, Li D (2011). Dielectrophoresis in microfluidics technology. Electrophoresis.

[28] Khoshmanesh K, Nahavandi S, Baratchi S, Mitchell A, Kalantar-zadeh K (2011). Dielectrophoretic platforms for bio-microfluidic systems. Biosens Bioelectron.

[29] Kuzyk A (2011). Dielectrophoresis at the nanoscale. Electrophoresis.

[30] Zhang C, Khoshmanesh K, Mitchell A, Kalantar-Zadeh K (2010). Dielectrophoresis for manipulation of micro/nano particles in microfluidic systems. Anal Bioanal Chem.

[31] Krupke R, Hennrich F, Lhneysen HV, Kappes MM (2003). Separation of metallic from semiconducting single-walled carbon nanotubes. Science.

[32] Chiou PY, Ohta AT, Wu MC (2005). Massively parallel manipulation of single cells and microparticles using optical images. Nature.

[33] Barik A, Zhang Y, Grassi R, Nadappuram BP, Edel JB, Low T (2017). Graphene-edge dielectrophoretic tweezers for trapping of biomolecules. Nat Commun.

[34] Tanaka T, Mizutani F, Yasukawa T (2016). Dielectrophoretic tweezers for pickup and relocation of individual cells using microdisk electrodes with a microcavity. Electrochemistry.

[35] Menachery A, Graham D, Messerli SM, Pethig R, Smith PJ (2011). Dielectrophoretic tweezer for isolating and manipulating target cells. IET Nanobiotechnol.

[36] Graham DM, Messerli MA, Pethig R (2012). Spatial manipulation of cells and organelles using single electrode dielectrophoresis. BioTechniques.

[37] Hunt TP, Westervelt RM (2006). Dielectrophoresis tweezers for single cell manipulation. Biomed Microdevices.

[38] Schnelle T, Mller T, Hagedorn R, Voigt A, Fuhr G (1999). Single micro electrode dielectrophoretic tweezers for manipulation of suspended cells and particles. BBA-Gen Subjects.

[39] Hirano K, Nagata H, Ishido T, Tanaka Y, Baba Y, Ishikawa M (2008). Sizing of single globular DNA molecules by using a circular acceleration technique with laser trapping. Anal Chem.

[40] Lei U, Sun PH, Pethig R (2011). Refinement of the theory for extracting cell dielectric properties from dielectrophoresis and electrorotation experiments. Biomicrofluidics.

[41] Sabuncu AC, Asmar AJ, Stacey MW, Beskok A (2015). Differential dielectric responses of chondrocyte and Jurkat cells in electromanipulation buffers. Electrophoresis.

[42] Pethig R, Talary MS (2007). Dielectrophoretic detection of membrane morphology changes in Jurkat T-cells undergoing etoposide-induced apoptosis. IET Nanobiotechnol.

[43] Tan Q, Ferrier GA, Chen BK, Wang C, Sun Y (2012). Quantification of the specific membrane capacitance of single cells using a microfluidic device and impedance spectroscopy measurement. Biomicrofluidics.

[44] Sano MB, Henslee EA, Schmelz E, Davalos RV (2011). Contactless dielectrophoretic spectroscopy: examination of the dielectric properties of cells found in blood. Electrophoresis.

[45] Zhou T, Ming Y, Perry SF, Tatic-Lucic S (2016). Estimation of the physical properties of neurons and glial cells using dielectrophoresis crossover frequency. J Biol Phys.

[46] Lynch BP, Hilton AM, Simpson GJ (2006). Nanoscale dielectrophoretic spectroscopy of individual immobilized mammalian blood cells. Biophys J.

[47] Beving H, Eriksson LEG, Davey CL, Kell DB (1994). Dielectric properties of human blood and erythrocytes at radio frequencies (0.210 MHz); dependence on cell volume fraction and medium composition. Eur Biophys J.

[48] Coley HM, Labeed FH, Thomas H, Hughes MP (2007). Biophysical characterization of MDR breast cancer cell lines reveals the cytoplasm is critical in determining drug sensitivity. BBA-Gen Subjects.

[49] Labeed FH, Coley HM, Hughes MP (2006). Differences in the biophysical properties of membrane and cytoplasm of apoptotic cells revealed using dielectrophoresis. BBA-Gen Subjects.

[50] Chin S, Hughes MP, Coley HM, Labeed FH (2006). Rapid assessment of early biophysical changes in K562 cells during apoptosis determined using dielectrophoresis. Int J Nanomedicine.

[51] Gimsa J, Mller T, Schnelle T, Fuhr G (1996). Dielectric spectroscopy of single human erythrocytes at physiological ionic strength: dispersion of the cytoplasm. Biophys J.

