# Supplementary material for: Frequency-Modulated Wave Dielectrophoresis of Vesicles And Cells: Periodic U-Turns at the Crossover Frequency
Source: Nanoscale Res Lett. 2018 Jun 7;13:169. doi: 10.1186/s11671-018-2583-5 (PMC5991112; doi:10.1186/s11671-018-2583-5)
Supplement: Supplementary file 2 — Derivation of Eqs. (8) and (9) in the wide band limit (WBL). We provide the details in obtaining the approximate expression of Eqs. (8) and (9). Our focus is on clarifying how the integral in Eq. (5) is reduced to a simple form in the WBL, as seen from Eqs. (A2), (A3) and (A10). (PDF 33 kb) [file 11671_2018_2583_MOESM2_ESM.pdf]

A. DERIVATION OF EQS. (8) AND (9) IN THE WIDE BAND LIMIT (WBL)

Outline

In Eq. (9), $\langle \boldsymbol{F}_{\mathrm{DEP}}(\boldsymbol{r},t)\rangle$ denotes the mean FM-DEP force that has been averaged over cycles of $\theta(t)$ in the FM field. The general expression of $\boldsymbol{F}_{\mathrm{DEP}}(\boldsymbol{r},t)$ prior to averaging is given by a set of Eqs. (3) to (5), which applies to the FM-DEP as well as the AC-DEP. It follows from Eqs. (3) to (5) that

$$\langle \boldsymbol{F}_{\mathrm{DEP}}(\boldsymbol{r},t)\rangle = 4\pi R^3 \epsilon_{\mathrm{out}} K_H \left[\langle \boldsymbol{E}\cdot\nabla\boldsymbol{E}\rangle + \frac{\tau}{\Delta\tau}\left\langle \widetilde{\boldsymbol{E}}\cdot\nabla\boldsymbol{E}\right\rangle \right], \tag{A1}$$

similarly to the AC-DEP force (see the first line on the right hand side of Eq. (6)), where K_H and $\Delta\tau$ are defined as follows: $K_H = (\epsilon_{\mathrm{in}}-\epsilon_{\mathrm{out}})/(\epsilon_{\mathrm{in}}+2\epsilon_{\mathrm{out}})$, and $\Delta\tau^{-1} = \tau_0^{-1} - \tau^{-1}$ using the radius R of the spherical object and two characteristic times of $\tau_0 = (\epsilon_{\mathrm{in}}-\epsilon_{\mathrm{out}})/(\sigma_{\mathrm{in}}-\sigma_{\mathrm{out}})$ and $\tau = (\epsilon_{\mathrm{in}}+2\epsilon_{\mathrm{out}})/(\sigma_{\mathrm{in}}+2\sigma_{\mathrm{out}})$. Substituting the expression (5) for $\widetilde{\boldsymbol{E}}$ into $\left\langle \widetilde{\boldsymbol{E}}\cdot\nabla\boldsymbol{E}\right\rangle$, we have

$$\left\langle \widetilde{\boldsymbol{E}}\cdot\nabla\boldsymbol{E}\right\rangle = \langle I(t)\cos\theta(t)\rangle\, \boldsymbol{A}\cdot\nabla\boldsymbol{A}, \tag{A2}$$

$$I(t) = \frac{1}{\tau}\int_0^t ds\, \cos\theta(t-s)\, e^{-s/\tau}. \tag{A3}$$

Integration of $I(t)$ by part provides that

$$\begin{aligned} I(t) &= \left[-\cos\theta(t-s)\, e^{-s/\tau}\right]_0^t + 2\pi\int_0^t ds\, f(t-s)\sin\theta(t-s)e^{-s/\tau} \\ &= \cos\theta(t) - e^{-t/\tau} - \left[2\pi\tau f(t-s)\sin\theta(t-s)\, e^{-s/\tau}\right]_0^t \\ &\quad -2\pi\tau\int_0^t ds\, \frac{df(t-s)}{ds}\sin\theta(t-s)e^{-s/\tau} - (2\pi)^2\tau\int_0^t ds\, f^2(t-s)\cos\theta(t-s)e^{-s/\tau} \\ &= \cos\theta(t) + 2\pi f(t)\tau\sin\theta(t) - e^{-t/\tau} - \{2\pi f(t)\}^2\,\tau\int_0^t ds\, g(t-s)e^{-s/\tau}, \end{aligned} \tag{A4}$$

where

$$f(t-s) = f_c + \Delta f\cos\{2\pi f_m(t-s)\}, \tag{A5}$$

$$\theta(t-s) = 2\pi\left[f_c + \Delta f\left(\frac{\sin\{2\pi f_m(t-s)\}}{2\pi f_m(t-s)}\right)\right](t-s), \tag{A6}$$

$$g(t-s) = \frac{f_m}{\Delta f}\left[\left(\frac{\Delta f}{f(t)}\right)^2 \sin\{2\pi f_m(t-s)\}\sin\theta(t-s)\right] + \left\{\frac{f(t-s)}{f(t)}\right\}^2\cos\theta(t-s) \tag{A7}$$

We will validate below the following approximation in the WBL:

$$g(t-s) \approx \cos\theta(t-s), \tag{A8}$$

so that the right hand side of Eq. (A4) can read

$$I(t) = \cos\theta(t) + 2\pi f(t)\tau \sin\theta(t) - e^{-t/\tau} - \{2\pi f(t)\tau\}^2 I(t). \tag{A9}$$

In other words, the WBL approximation yields

$$I(t) = \frac{1}{1+\{2\pi f(t)\tau\}^2}\left\{\cos\theta(t) + 2\pi f(t)\tau\sin\theta(t) - e^{-t/\tau}\right\}. \tag{A10}$$

Equation (A2) indicates that the obtained function $I(t)$ multiplied by $\cos\theta(t)$ needs to be further averaged over the cycles with respect to $\theta(t)$. As a consequence, there remains only the term including $\langle\cos^2\theta(t)\rangle = 1/2$ and we have

$$\langle I(t)\cos\theta(t)\rangle = \frac{1}{1+\{2\pi f(t)\tau\}^2}\left(\frac{1}{2}\right). \tag{A11}$$

Equation (8) given in the main text is thus derived by plugging Eq. (A11) into Eq. (A2) as follows:

$$\begin{aligned}\left\langle \widetilde{\boldsymbol{E}}\cdot\nabla\boldsymbol{E}\right\rangle &= \frac{1}{1+\{2\pi f(t)\tau\}^2}\left(\frac{\boldsymbol{A}\cdot\nabla\boldsymbol{A}}{2}\right)\\ &= \frac{1}{1+\{2\pi f(t)\tau\}^2}\left(\frac{\nabla\boldsymbol{A}_{\mathrm{RMS}}^2}{2}\right),\end{aligned} \tag{A12}$$

where use has been made of the relation: $\boldsymbol{A}\cdot\nabla\boldsymbol{A} = \nabla\boldsymbol{A}^2/2 = \nabla\boldsymbol{A}_{\mathrm{RMS}}^2$. Substituting Eq. (A12) into Eq. (A1), we have

$$\begin{aligned}\langle\boldsymbol{F}_{\mathrm{DEP}}(\boldsymbol{r},t)\rangle &= 4\pi R^3\epsilon_{\mathrm{out}}K_H\left[\frac{\nabla\boldsymbol{A}_{\mathrm{RMS}}^2}{2} + \frac{\tau}{\Delta\tau}\left\{\frac{1}{1+\{2\pi f(t)\tau\}^2}\left(\frac{\nabla\boldsymbol{A}_{\mathrm{RMS}}^2}{2}\right)\right\}\right],\\ &= 4\pi R^3\epsilon_{\mathrm{out}}K_H\left[\frac{\tau}{\tau_0}\left\{\frac{1}{1+\{2\pi f(t)\tau\}^2}\left(\frac{\nabla\boldsymbol{A}_{\mathrm{RMS}}^2}{2}\right)\right\} + \frac{\nabla\boldsymbol{A}_{\mathrm{RMS}}^2}{2}\left(1 - \frac{1}{1+\{2\pi f(t)\tau\}^2}\right)\right],\\ &= 2\pi R^3\epsilon_{\mathrm{out}}\left[K_L\frac{1}{1+\{2\pi f(t)\tau\}^2} + K_H\frac{\{2\pi f(t)\tau\}^2}{1+\{2\pi f(t)\tau\}^2}\right]\nabla\boldsymbol{A}_{\mathrm{RMS}}^2,\end{aligned} \tag{A13}$$

where $K_L = K_H\tau/\tau_0 = (\sigma_{\mathrm{in}} - \sigma_{\mathrm{out}})/(\sigma_{\mathrm{in}} + 2\sigma_{\mathrm{out}})$. Setting that

$$\chi\{f(t)\} = \frac{2\pi R^3\epsilon_{\mathrm{out}}}{1+\{2\pi f(t)\tau\}^2}\left[K_L + \{2\pi f(t)\tau\}^2 K_H\right], \tag{A14}$$

Eq. (9) is verified.

From Eq. (A7) to Eq. (A8) in the WBL

To complete the derivation of Eq. (8) in the main text, the WBL approximation (A8) of Eq. (A7) remains to be verified. It is first to be noted that the first term on the right hand side of Eq. (A7) is negligible:

$$g(t-s) \approx \left\{ \frac{f(t-s)}{f(t)} \right\}^2 \cos\theta(t-s). \tag{A15}$$

The above approximation (A15) is validated from the the experimental condition of $\Delta f < f_c - \Delta f$ (or, $\Delta f / f(t) < f(t-s)/f(t)$) as well as the WBL condition of $f_m/\Delta f \ll 1$. Furthermore, we would like to prove that

$$\begin{aligned} \left\{ \frac{f(t-s)}{f(t)} \right\}^2 \cos\theta(t-s) &= \cos\theta(t-s) + \left\{ \frac{\{f(t-s)-f(t)\}\cos\theta(t-s)}{f(t)} \right\}^2 \frac{1}{\cos\theta(t-s)} \\ &\approx \cos\theta(t-s), \end{aligned} \tag{A16}$$

implying that the WBL condition leads to

$$\begin{aligned} \frac{\{f(t-s)-f(t)\}\cos\theta(t-s)}{f(t)} &= \frac{\Delta f}{f(t)} \{\cos 2\pi f_m(t-s)\cos\theta(t-s) - \cos 2\pi f_m(t)\cos\theta(t-s)\} \\ &\approx 0. \end{aligned} \tag{A17}$$

To show this, we consider the transformed expression as follows:

$$\begin{aligned} &\cos\{2\pi f_m(t-s)\}\cos\theta(t-s) - \cos(2\pi f_m t)\cos\theta(t-s) \\ &\quad = \cos(2\pi f_m t)\{\cos(2\pi f_m s)\cos\theta(t-s) - \cos\theta(t-s)\} - \sin(2\pi f_m t)\sin(2\pi f_m s)\cos\theta(t-s) \\ &\quad = \cos(2\pi f_m t)\left\{ \frac{\cos\beta_+ + \cos\beta_-}{2} - \cos\theta(t-s) \right\} - \sin(2\pi f_m t)\left(\frac{\sin\beta_+ + \sin\beta_-}{2} \right), \end{aligned} \tag{A18}$$

where

$$\begin{aligned} \beta_\pm &= 2\pi f_m s \pm \theta(t-s) \\ &= 2\pi f_c \left[\frac{f_m}{f_c} s \pm \left\{ 1 + \frac{\Delta f}{f_c} \left(\frac{\sin\{2\pi f_m(t-s)\}}{2\pi f_m(t-s)} \right) \right\} (t-s) \right]. \end{aligned} \tag{A19}$$

In the WBL of $f_m/f_c \ll 1$, we have $\beta_\pm \approx \pm\theta(t-s)$, thereby yielding

$$\begin{aligned} \frac{\cos\beta_+ + \cos\beta_-}{2} &\approx \cos\theta(t-s), \\ \frac{\sin\beta_+ + \sin\beta_-}{2} &\approx 0. \end{aligned} \tag{A20}$$

Combining Eq. (A18) with the above approximations of Eq. (A20), we verify that

$$\cos\{2\pi f_m(t-s)\}\cos\theta(t-s) - \cos(2\pi f_m t)\cos\theta(t-s) \approx 0; \tag{A21}$$

Equation (A17) (or, Eq. (A16)) is thus validated. To summrize, we have confirmed, from proving Eqs. (A15) and (A16), the approximation that Eq. (A7) is reduced to Eq. (A8) in the WBL.
